# Rötheln

**Published:** 1875-09

**Authors:** J. C. Bishop

**Affiliations:** Middleport, Ohio


### "RÖTHELN."

BY J. C. BISHOP, M. D., MIDDLEPORT, OHIO.

The disease now recognized as Rötheln was described over fifty years ago by the German physicians; but it attracted such little attention from the profession of other nations, that, until within the last few years, we have heard but little of it; while, in fact, it has existed all the while, but been improperly considered under the head of mild Scarlatina or Measles, because of its resemblance to these affections. Rötheln is, according to Dunglison, synonymous with the terms Rubella, Rosalia, Rubeola Northa, Erythema Scarlatiniforme, Bostarc or German Measles, Scarlatina Morbillosa, Hybrid of Measles and Scarlatina and Anomalous Exanthem. "Is a form of eruption, resembling measles, sometimes not unlike Scarlatina; dusky red, uniformly distributed, without catarrhal symptoms, not contagious, and of very favorable prognosis; by some regarded as Coexistent Measles and Scarlatina, was at first confounded with Variola, was called Rubeola by Hildenbrand." According to our observation and research, Prof. Dunglison is not strictly correct in this description of the disease in excluding catarrhal symptoms, as there are generally present more or less symptoms of this character in the disease. In April, 1870, Dr. Murchison reported two cases then in the Middlesex Hospital. In the same institution Dr. Robert Liveing reported (*Lancet*, March 14, 1874,) as having in June, 1873, five cases of the same disease. Dr. J. Lewis Smith published in the *Sanitarian*, July, 1874, an account of the disease which was then visiting New York, forty-eight cases having appeared in twenty-one families. The following case, taken from a clinical lecture by Dr. Liveing, in the Middlesex Hospital, is so nearly typical, that it is appropriated with due acknowledgments:

"Jane B——, aged 27, admitted June 10, 1873, (housemaid.) When about eight years old, the patient had a sharp attack of measles. On Sunday morning, the 8th day of June, at 7 a. m., she had a feeling of nausea, which lasted during the morning, but passed off after dinner. On Monday morning, the 9th, about 7

o'clock, the same feeling returned, accompanied with headache. At breakfast she found that her throat was sore on swallowing, and also noticed lumbar pains. About 2 p. m. she saw that her face was red, and a little swollen, then that her neck, back and chest were flushed in a similar manner.

"She went to a doctor the same evening, who stated that in his opinion her complaint was measles. On the 10th she noticed that her legs were affected, being red all over. She felt feverish. On admission, the whole body of the patient was covered with small patches of a red papular eruption, most marked on the back, where it was more or less confluent. There was slight sore throat, and the fauces were red. The conjunctivæ were somewhat suffused; the tongue was marked by the teeth, and of a brownish color in the center. The physical signs of the chest were fairly good; the bowels rather confined. The urine contained no albumen. June 12th. The rash is beginning to fade, and is slight on the hands and feet. 13th. The tonsils are rather swollen, the left more than the right. There is a slight trace of albumen in the urine. 14th. The left tonsil is ragged and painful; the vessels can be distinctly seen running over the posterior surface of the pharynx, which has scattered over it patches of a whitish yellow color; the rash is fading, but has not quite disappeared, and the temperature is normal. The bowels open freely. 16th. The urine is acid 10:32, and loaded with urates; no albumen. 17th. The throat is rather better, and there is no pain, and no rash to be seen. 25th. The patient was discharged convalescent. The eruption lasted seven days, but never became thoroughly confluent. The patient was under observation for some weeks after her discharge; there was a very slight brawny desquamation of cuticle." That Rötheln exists as an individual affection seems well established, and that it is neither measles nor scarlatina, in any sense, is equally true, from the fact that persons having had one or both the above named diseases, have no immunity from attacks of Rötheln; in the cases reported by Liveing, op. cit., all had previously had measles with one exception. Neither does Rötheln protect patients from subsequent attacks of measles or scarlatina; nor will it produce either measles or scarlatina in others, but will always propagate itself alone. If this be true, which observation has demonstrated, it will readily be conceded that Rötheln is a disease per se, as certainly as either the allied affections, and at the same time characterized by its own peculiar phenomena. However, like most other eruptive diseases, some of the manifestations may be absent; and indeed but few cases will appear in which all the symptoms are perfectly well marked. Yet, generally we have the premonitory fever, like that of measles, but of shorter duration, lasting on an average about 24 hours, while in measles it is of from three to four days. There is headache, pains in the lumbar region, sore throat, nausea and occasionally vomiting, with the appearance of a red papular eruption; and here we will find the conditions which will enable us to differentiate between this and measles and scarlatina. The eruption occurs in irregular patches, without a tendency to become crecentic in shape, as in measles; occasionally there is tolerably complete coalescence of the patches, and the skin assumes a brighter red and the eruption lasts longer than in either the last named disease or scarlatina. While in these diseases there is a peculiar tendency of the eruption to become profuse in the axilla and groin, in Rötheln entire absence of the eruption in these situations is the rule, and should a case present, accompanied by all the characteristics of measles or scarlatina, yet with an absence, according to some German author, as stated by Dr. A. L. Knight, of eruption in these situations, we would unhesitatingly diagnose Rötheln. Desquamation does not always take place, and when it does, it is not so extensive as in scarlatina. Authors differ somewhat as to the contagiousness and non-contagiousness of this disease, for as above stated, Dunglison says it is not contagious, while Liveing, without declaring it so, says it is far less contagious than either measles or scarlatina, but more distinctly epidemic. Smith conclues that it is "a contagious exanthematous fever, allied to measles and scarlet fever, but totally distinct from either." We have no doubt that the disease may be propagated just as all such exanthematous diseases are propagated, only requiring suitable conditions for its development. It is our firm conviction that numerous cases are treated annually in different localities, as either mild scartalina or measles, which really are neither the one nor the other, but in fact Rötheln. The treatment required is generally a "severe letting alone," in the ordinary cases, yet occasionally it might be necessary to employ a local application to the tonsils, and if necessary a saline cathartic may be administered. The only sequellæ which is of any gravity, is the Dropsy, which would depend upon a chronic albuminuria, but such a condition will be extremely rare.

June 18, 1875.

